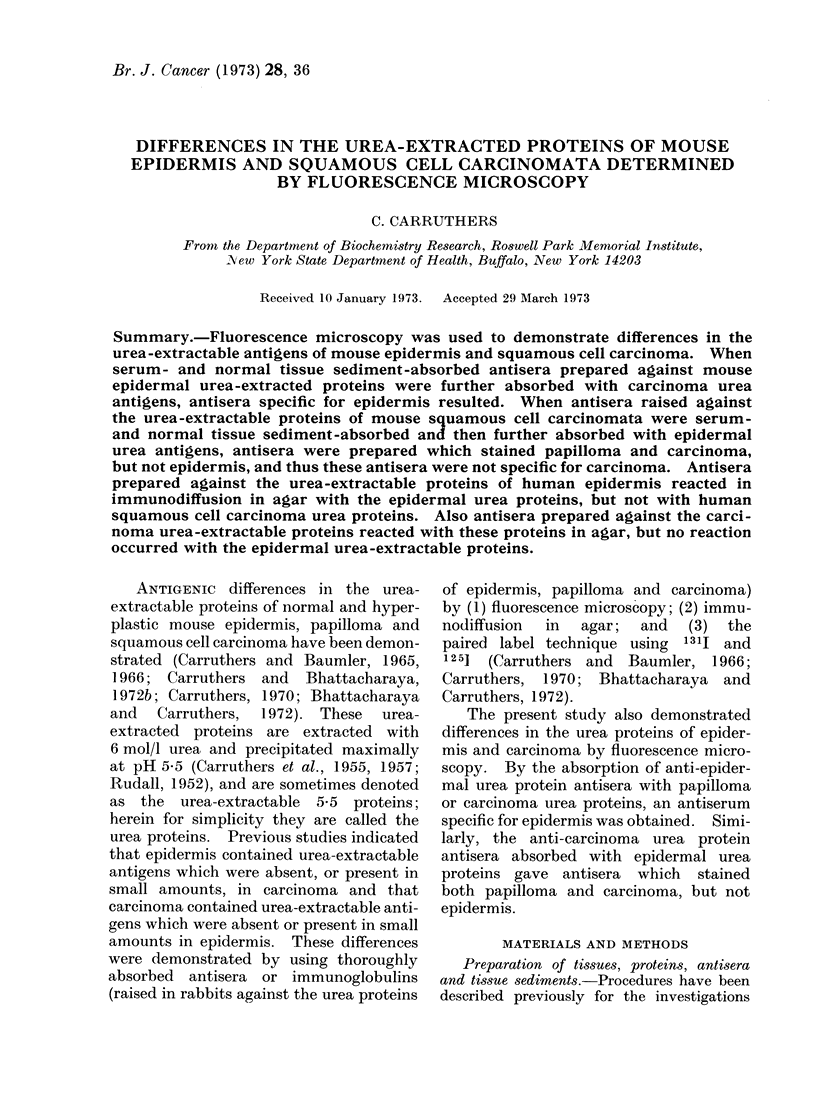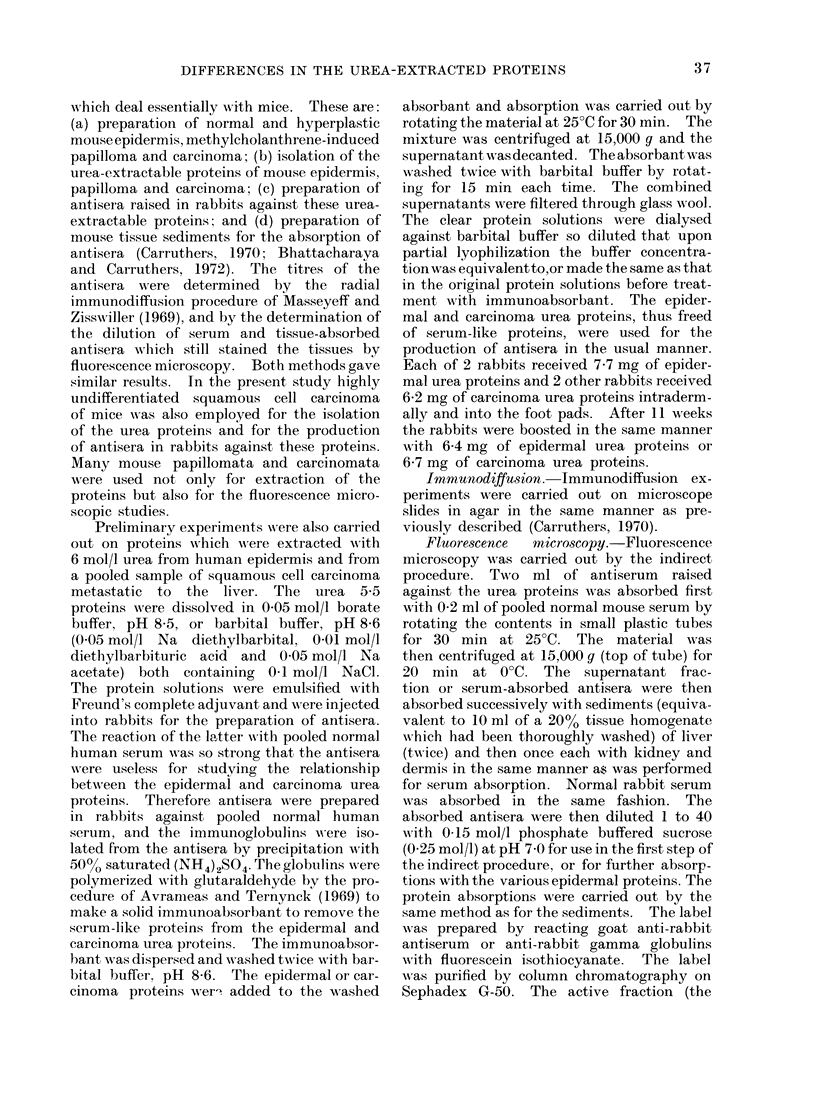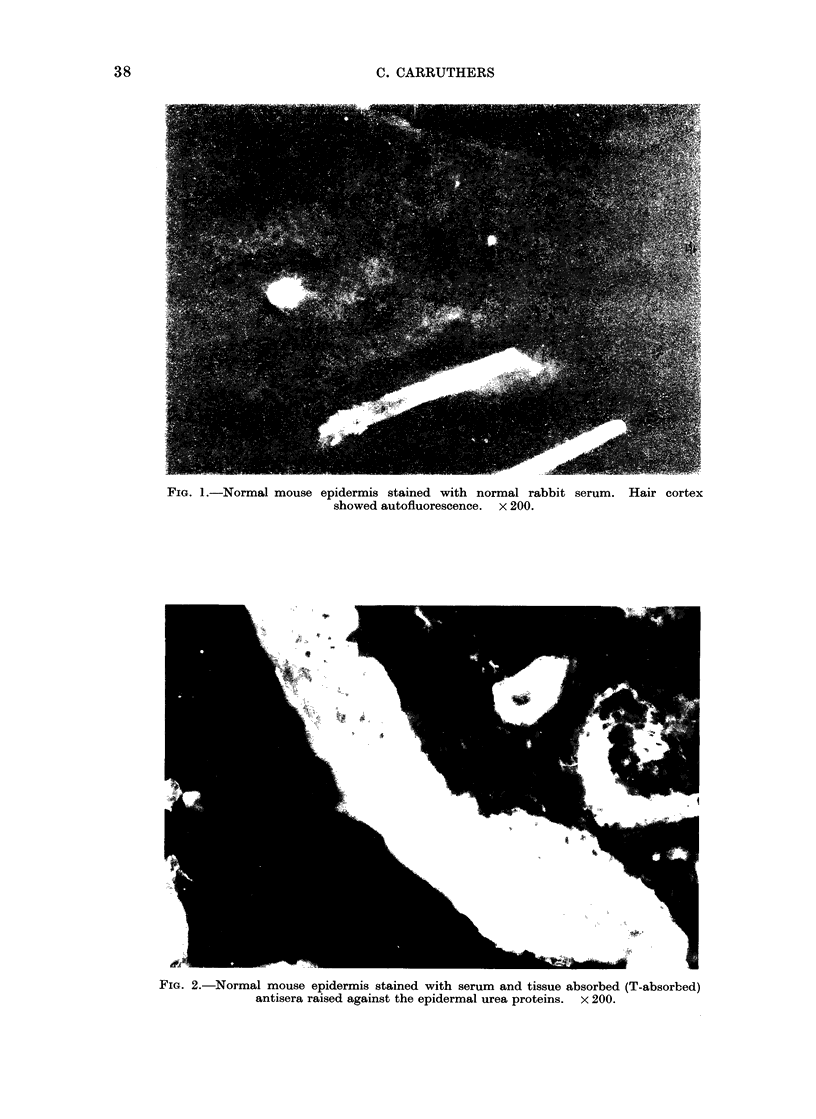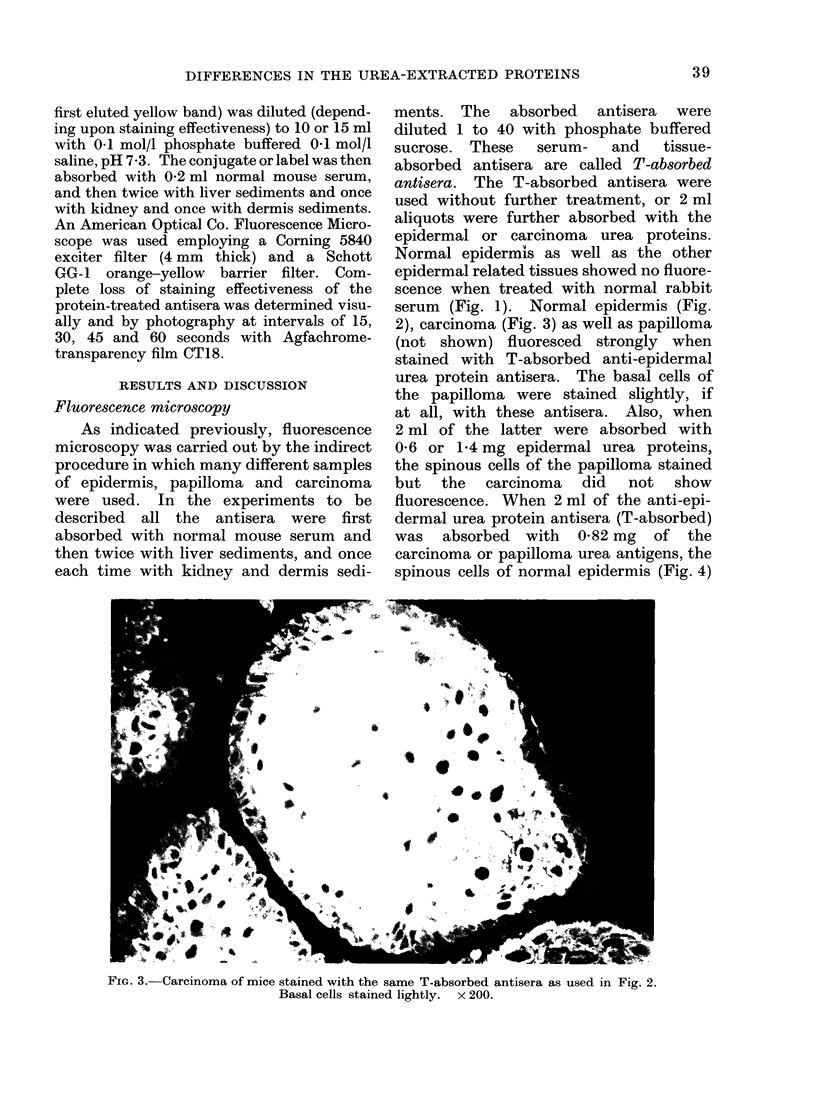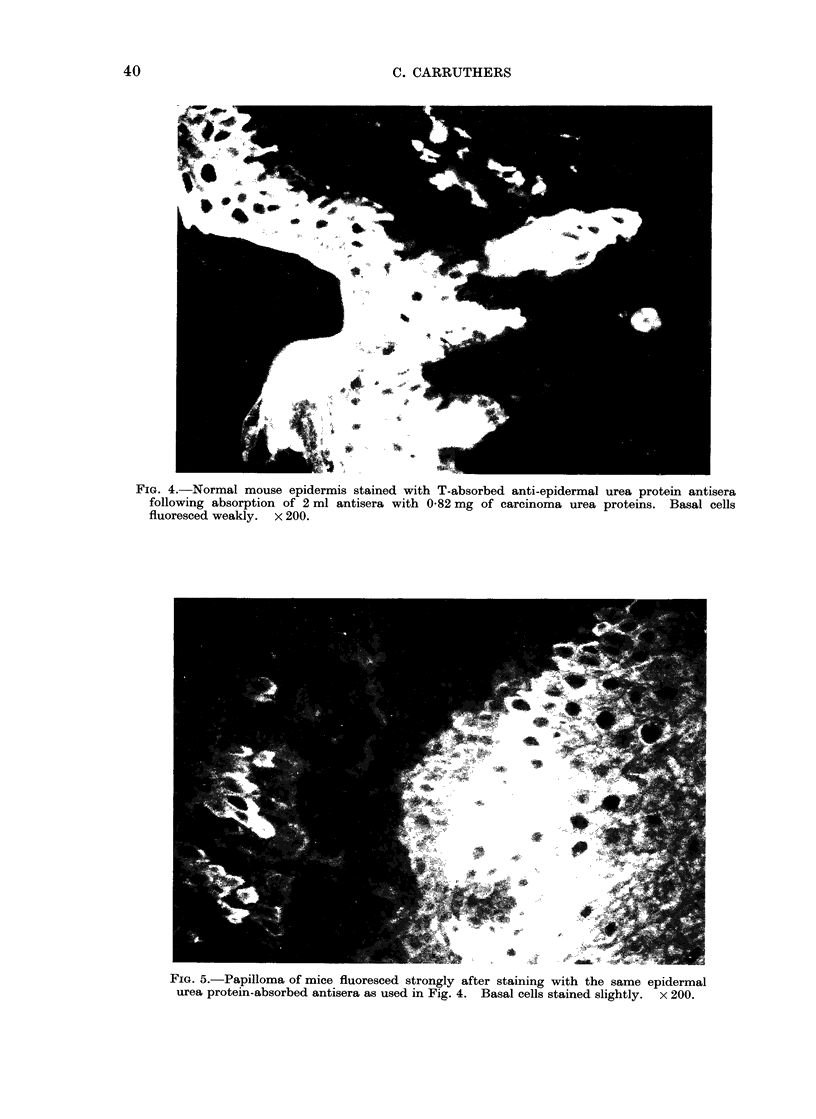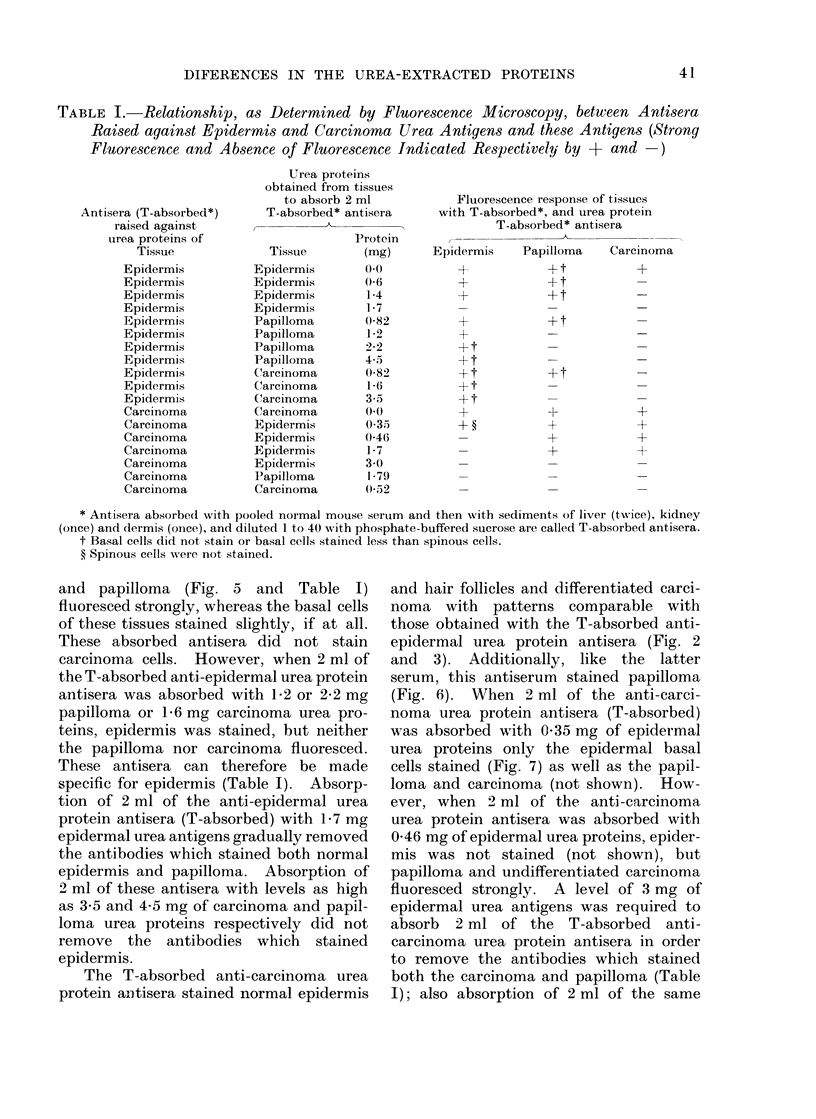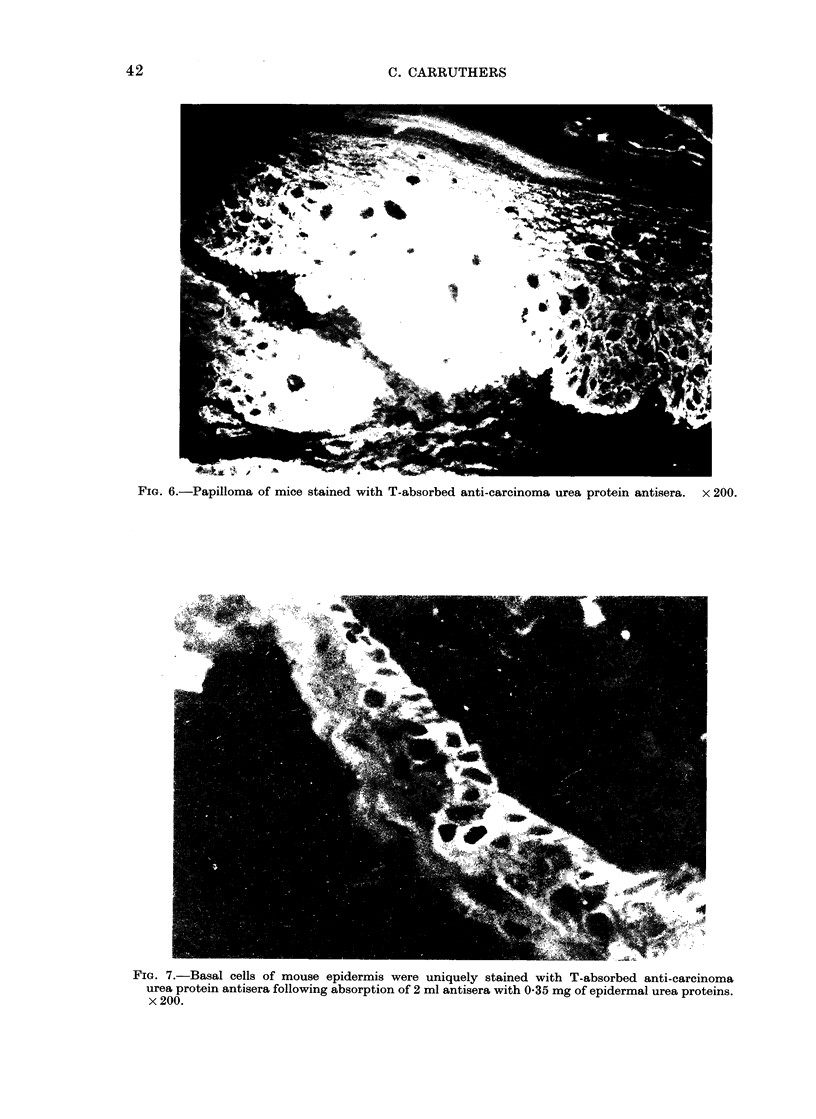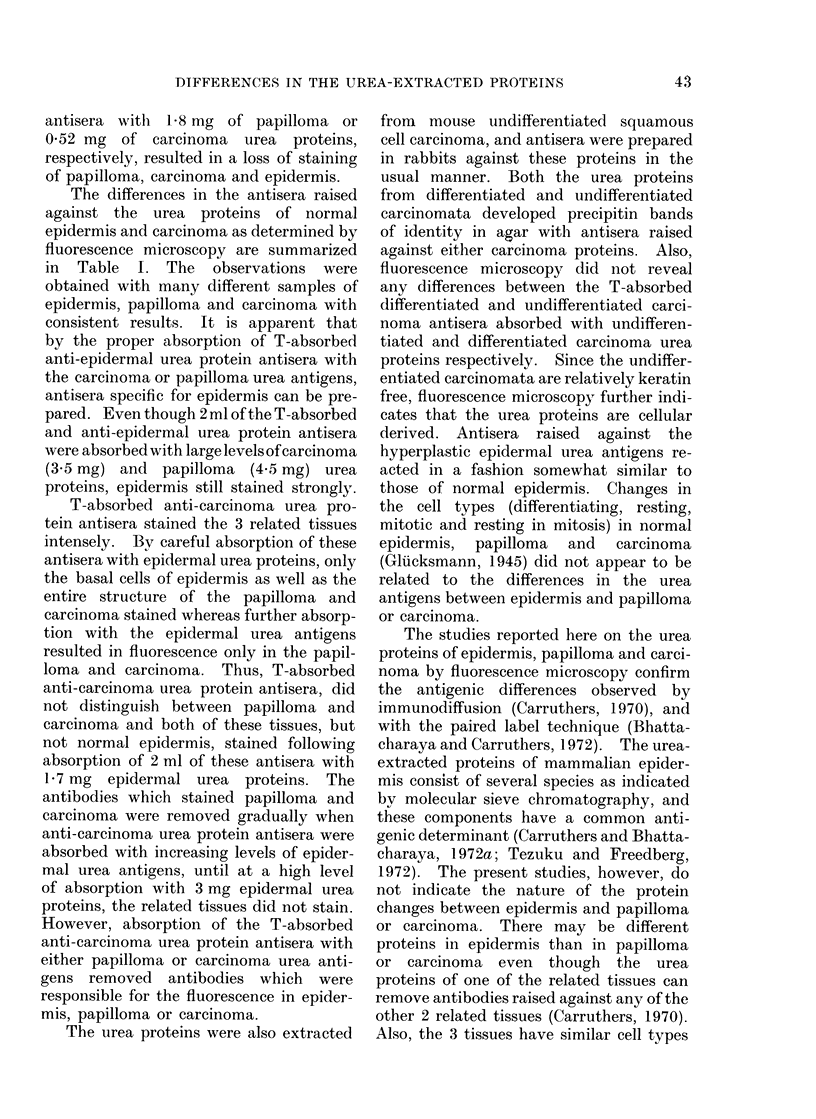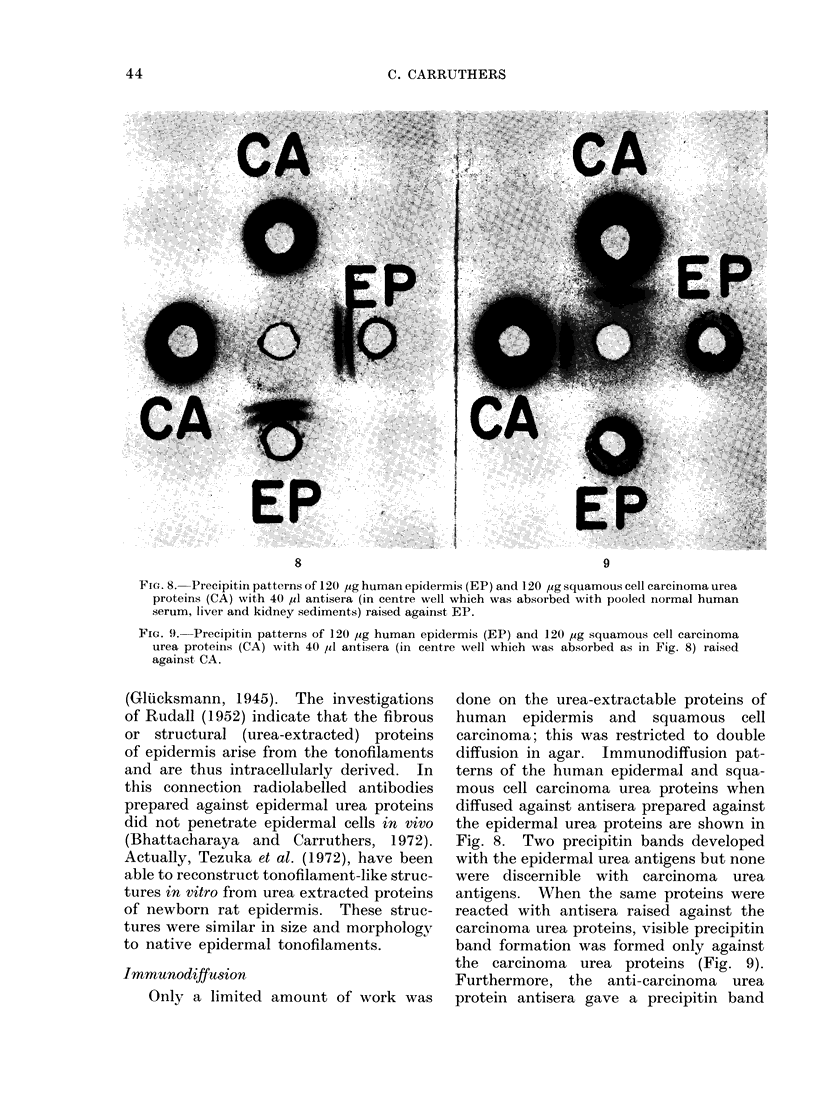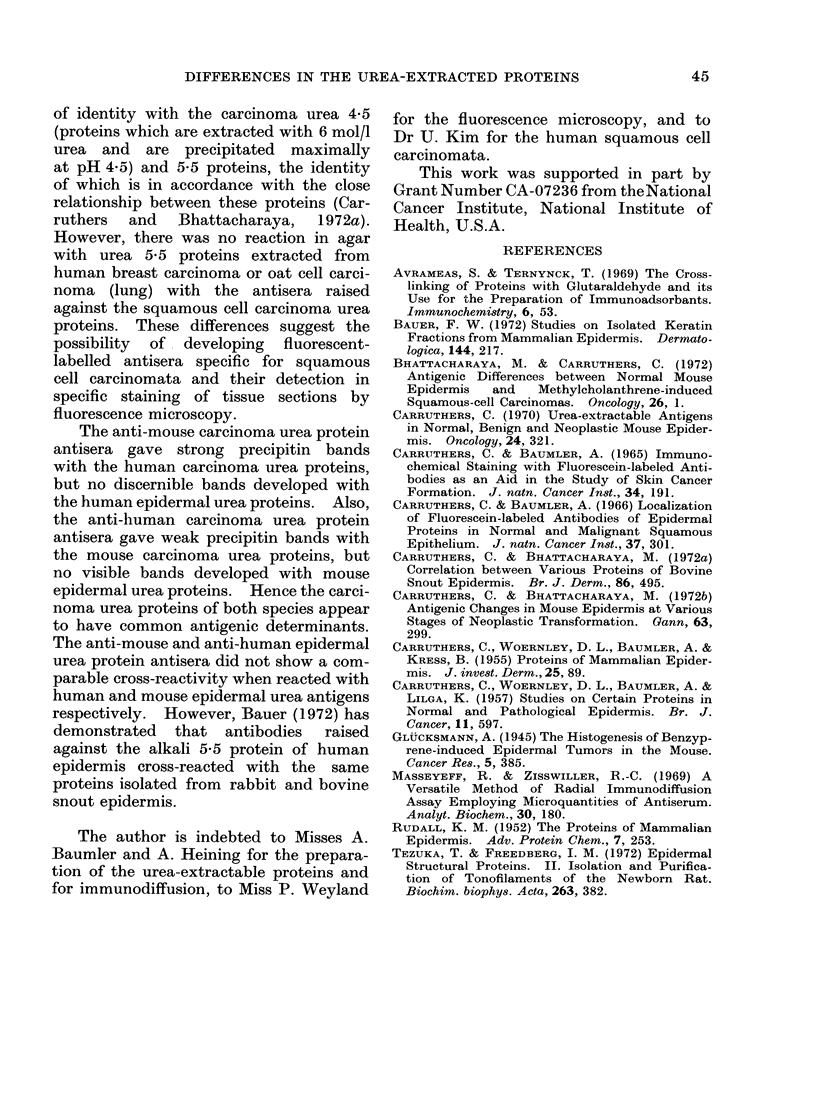# Differences in the Urea-extracted Proteins of Mouse Epidermis and Squamous-cell Carcinomata Determined by Fluorescence Microscopy

**DOI:** 10.1038/bjc.1973.68

**Published:** 1973-07

**Authors:** C. Carruthers

## Abstract

**Images:**


Br. J. Cancer (1973) 28, 36

### DIFFERENCES IN THE UREA-EXTRACTED PROTEINS OF MOUSE EPIDERMIS AND SQUAMOUS CELL CARCINOMATA DETERMINED BY FLUORESCENCE MICROSCOPY

C. CARRUTHERS

*From the Department of Biochemistry Research, Roswell Park Memorial Institute, New York State Department of Health, Buffalo, New York 14203*

Received 10 January 1973. Accepted 29 March 1973

**Summary.—Fluorescence microscopy was used to demonstrate differences in the urea-extractable antigens of mouse epidermis and squamous cell carcinoma. When serum- and normal tissue sediment-absorbed antisera prepared against mouse epidermal urea-extracted proteins were further absorbed with carcinoma urea antigens, antisera specific for epidermis resulted. When antisera raised against the urea-extractable proteins of mouse squamous cell carcinomata were serum- and normal tissue sediment-absorbed and then further absorbed with epidermal urea antigens, antisera were prepared which stained papilloma and carcinoma, but not epidermis, and thus these antisera were not specific for carcinoma. Antisera prepared against the urea-extractable proteins of human epidermis reacted in immunodiffusion in agar with the epidermal urea proteins, but not with human squamous cell carcinoma urea proteins. Also antisera prepared against the carcinoma urea-extractable proteins reacted with these proteins in agar, but no reaction occurred with the epidermal urea-extractable proteins.**

ANTIGENIC differences in the urea-extractable proteins of normal and hyperplastic mouse epidermis, papilloma and squamous cell carcinoma have been demonstrated (Carruthers and Baumler, 1965, 1966; Carruthers and Bhattacharaya, 1972*b*; Carruthers, 1970; Bhattacharaya and Carruthers, 1972). These urea-extracted proteins are extracted with 6 mol/l urea and precipitated maximally at pH 5·5 (Carruthers *et al.*, 1955, 1957; Rudall, 1952), and are sometimes denoted as the urea-extractable 5·5 proteins; herein for simplicity they are called the urea proteins. Previous studies indicated that epidermis contained urea-extractable antigens which were absent, or present in small amounts, in carcinoma and that carcinoma contained urea-extractable antigens which were absent or present in small amounts in epidermis. These differences were demonstrated by using thoroughly absorbed antisera or immunoglobulins (raised in rabbits against the urea proteins of epidermis, papilloma and carcinoma) by (1) fluorescence microscopy; (2) immunodiffusion in agar; and (3) the paired label technique using $^{131}I$ and $^{125}I$ (Carruthers and Baumler, 1966; Carruthers, 1970; Bhattacharaya and Carruthers, 1972).

The present study also demonstrated differences in the urea proteins of epidermis and carcinoma by fluorescence microscopy. By the absorption of anti-epidermal urea protein antisera with papilloma or carcinoma urea proteins, an antiserum specific for epidermis was obtained. Similarly, the anti-carcinoma urea protein antisera absorbed with epidermal urea proteins gave antisera which stained both papilloma and carcinoma, but not epidermis.

### MATERIALS AND METHODS

*Preparation of tissues, proteins, antisera and tissue sediments.*—Procedures have been described previously for the investigations which deal essentially with mice. These are: (a) preparation of normal and hyperplastic mouse epidermis, methylcholanthrene-induced papilloma and carcinoma; (b) isolation of the urea-extractable proteins of mouse epidermis, papilloma and carcinoma; (c) preparation of antisera raised in rabbits against these urea-extractable proteins; and (d) preparation of mouse tissue sediments for the absorption of antisera (Carruthers, 1970; Bhattacharaya and Carruthers, 1972). The titres of the antisera were determined by the radial immunodiffusion procedure of Masseyeff and Zisswiller (1969), and by the determination of the dilution of serum and tissue-absorbed antisera which still stained the tissues by fluorescence microscopy. Both methods gave similar results. In the present study highly undifferentiated squamous cell carcinoma of mice was also employed for the isolation of the urea proteins and for the production of antisera in rabbits against these proteins. Many mouse papillomata and carcinomata were used not only for extraction of the proteins but also for the fluorescence microscopic studies.

Preliminary experiments were also carried out on proteins which were extracted with 6 mol/l urea from human epidermis and from a pooled sample of squamous cell carcinoma metastatic to the liver. The urea 5·5 proteins were dissolved in 0·05 mol/l borate buffer, pH 8·5, or barbital buffer, pH 8·6 (0·05 mol/l Na diethylbarbital, 0·01 mol/l diethylbarbituric acid and 0·05 mol/l Na acetate) both containing 0·1 mol/l NaCl. The protein solutions were emulsified with Freund's complete adjuvant and were injected into rabbits for the preparation of antisera. The reaction of the latter with pooled normal human serum was so strong that the antisera were useless for studying the relationship between the epidermal and carcinoma urea proteins. Therefore antisera were prepared in rabbits against pooled normal human serum, and the immunoglobulins were isolated from the antisera by precipitation with 50% saturated $(NH_4)_2SO_4$. The globulins were polymerized with glutaraldehyde by the procedure of Avrameas and Ternynck (1969) to make a solid immunoabsorbant to remove the serum-like proteins from the epidermal and carcinoma urea proteins. The immunoabsorbant was dispersed and washed twice with barbital buffer, pH 8·6. The epidermal or carcinoma proteins were added to the washed absorbant and absorption was carried out by rotating the material at 25°C for 30 min. The mixture was centrifuged at 15,000 *g* and the supernatant was decanted. The absorbant was washed twice with barbital buffer by rotating for 15 min each time. The combined supernatants were filtered through glass wool. The clear protein solutions were dialysed against barbital buffer so diluted that upon partial lyophilization the buffer concentration was equivalent to, or made the same as that in the original protein solutions before treatment with immunoabsorbant. The epidermal and carcinoma urea proteins, thus freed of serum-like proteins, were used for the production of antisera in the usual manner. Each of 2 rabbits received 7·7 mg of epidermal urea proteins and 2 other rabbits received 6·2 mg of carcinoma urea proteins intradermally and into the foot pads. After 11 weeks the rabbits were boosted in the same manner with 6·4 mg of epidermal urea proteins or 6·7 mg of carcinoma urea proteins.

*Immunodiffusion.*—Immunodiffusion experiments were carried out on microscope slides in agar in the same manner as previously described (Carruthers, 1970).

*Fluorescence microscopy.*—Fluorescence microscopy was carried out by the indirect procedure. Two ml of antiserum raised against the urea proteins was absorbed first with 0·2 ml of pooled normal mouse serum by rotating the contents in small plastic tubes for 30 min at 25°C. The material was then centrifuged at 15,000 *g* (top of tube) for 20 min at 0°C. The supernatant fraction or serum-absorbed antisera were then absorbed successively with sediments (equivavalent to 10 ml of a 20% tissue homogenate which had been thoroughly washed) of liver (twice) and then once each with kidney and dermis in the same manner as was performed for serum absorption. Normal rabbit serum was absorbed in the same fashion. The absorbed antisera were then diluted 1 to 40 with 0·15 mol/l phosphate buffered sucrose (0·25 mol/l) at pH 7·0 for use in the first step of the indirect procedure, or for further absorptions with the various epidermal proteins. The protein absorptions were carried out by the same method as for the sediments. The label was prepared by reacting goat anti-rabbit antiserum or anti-rabbit gamma globulins with fluorescein isothiocyanate. The label was purified by column chromatography on Sephadex G-50. The active fraction (the

FIG. 1.—Normal mouse epidermis stained with normal rabbit serum. Hair cortex showed autofluorescence. ×200.

FIG. 2.—Normal mouse epidermis stained with serum and tissue absorbed (T-absorbed) antisera raised against the epidermal urea proteins. ×200.

first eluted yellow band) was diluted (depending upon staining effectiveness) to 10 or 15 ml with 0·1 mol/l phosphate buffered 0·1 mol/l saline, pH 7·3. The conjugate or label was then absorbed with 0·2 ml normal mouse serum, and then twice with liver sediments and once with kidney and once with dermis sediments. An American Optical Co. Fluorescence Microscope was used employing a Corning 5840 exciter filter (4 mm thick) and a Schott GG-1 orange–yellow barrier filter. Complete loss of staining effectiveness of the protein-treated antisera was determined visually and by photography at intervals of 15, 30, 45 and 60 seconds with Agfachrome-transparency film CT18.

### RESULTS AND DISCUSSION

### *Fluorescence microscopy*

As indicated previously, fluorescence microscopy was carried out by the indirect procedure in which many different samples of epidermis, papilloma and carcinoma were used. In the experiments to be described all the antisera were first absorbed with normal mouse serum and then twice with liver sediments, and once each time with kidney and dermis sediments. The absorbed antisera were diluted 1 to 40 with phosphate buffered sucrose. These serum- and tissue-absorbed antisera are called *T-absorbed antisera*. The T-absorbed antisera were used without further treatment, or 2 ml aliquots were further absorbed with the epidermal or carcinoma urea proteins. Normal epidermis as well as the other epidermal related tissues showed no fluorescence when treated with normal rabbit serum (Fig. 1). Normal epidermis (Fig. 2), carcinoma (Fig. 3) as well as papilloma (not shown) fluoresced strongly when stained with T-absorbed anti-epidermal urea protein antisera. The basal cells of the papilloma were stained slightly, if at all, with these antisera. Also, when 2 ml of the latter were absorbed with 0·6 or 1·4 mg epidermal urea proteins, the spinous cells of the papilloma stained but the carcinoma did not show fluorescence. When 2 ml of the anti-epidermal urea protein antisera (T-absorbed) was absorbed with 0·82 mg of the carcinoma or papilloma urea antigens, the spinous cells of normal epidermis (Fig. 4)

FIG. 3.—Carcinoma of mice stained with the same T-absorbed antisera as used in Fig. 2. Basal cells stained lightly. ×200.

FIG. 4.—Normal mouse epidermis stained with T-absorbed anti-epidermal urea protein antisera following absorption of 2 ml antisera with 0·82 mg of carcinoma urea proteins. Basal cells fluoresced weakly. ×200.

FIG. 5.—Papilloma of mice fluoresced strongly after staining with the same epidermal urea protein-absorbed antisera as used in Fig. 4. Basal cells stained slightly. ×200.

TABLE I.—*Relationship, as Determined by Fluorescence Microscopy, between Antisera Raised against Epidermis and Carcinoma Urea Antigens and these Antigens (Strong Fluorescence and Absence of Fluorescence Indicated Respectively by + and —)*

| Antisera (T-absorbed*) raised against urea proteins of Tissue | Urea proteins obtained from tissues to absorb 2 ml T-absorbed* antisera | | Fluorescence response of tissues with T-absorbed*, and urea protein T-absorbed* antisera | | |
|---|---|---|---|---|---|
| | Tissue | Protein (mg) | Epidermis | Papilloma | Carcinoma |
| Epidermis | Epidermis | 0·0 | + | +† | + |
| Epidermis | Epidermis | 0·6 | + | +† | — |
| Epidermis | Epidermis | 1·4 | + | +† | — |
| Epidermis | Epidermis | 1·7 | — | — | — |
| Epidermis | Papilloma | 0·82 | + | +† | — |
| Epidermis | Papilloma | 1·2 | + | — | — |
| Epidermis | Papilloma | 2·2 | +† | — | — |
| Epidermis | Papilloma | 4·5 | +† | — | — |
| Epidermis | Carcinoma | 0·82 | +† | +† | — |
| Epidermis | Carcinoma | 1·6 | +† | — | — |
| Epidermis | Carcinoma | 3·5 | +† | — | — |
| Carcinoma | Carcinoma | 0·0 | + | + | + |
| Carcinoma | Epidermis | 0·35 | + § | + | + |
| Carcinoma | Epidermis | 0·46 | — | + | + |
| Carcinoma | Epidermis | 1·7 | — | + | + |
| Carcinoma | Epidermis | 3·0 | — | — | — |
| Carcinoma | Papilloma | 1·79 | — | — | — |
| Carcinoma | Carcinoma | 0·52 | — | — | — |

* Antisera absorbed with pooled normal mouse serum and then with sediments of liver (twice), kidney (once) and dermis (once), and diluted 1 to 40 with phosphate-buffered sucrose are called T-absorbed antisera.
† Basal cells did not stain or basal cells stained less than spinous cells.
§ Spinous cells were not stained.

and papilloma (Fig. 5 and Table I) fluoresced strongly, whereas the basal cells of these tissues stained slightly, if at all. These absorbed antisera did not stain carcinoma cells. However, when 2 ml of the T-absorbed anti-epidermal urea protein antisera was absorbed with 1·2 or 2·2 mg papilloma or 1·6 mg carcinoma urea proteins, epidermis was stained, but neither the papilloma nor carcinoma fluoresced. These antisera can therefore be made specific for epidermis (Table I). Absorption of 2 ml of the anti-epidermal urea protein antisera (T-absorbed) with 1·7 mg epidermal urea antigens gradually removed the antibodies which stained both normal epidermis and papilloma. Absorption of 2 ml of these antisera with levels as high as 3·5 and 4·5 mg of carcinoma and papilloma urea proteins respectively did not remove the antibodies which stained epidermis.

The T-absorbed anti-carcinoma urea protein antisera stained normal epidermis and hair follicles and differentiated carcinoma with patterns comparable with those obtained with the T-absorbed anti-epidermal urea protein antisera (Fig. 2 and 3). Additionally, like the latter serum, this antiserum stained papilloma (Fig. 6). When 2 ml of the anti-carcinoma urea protein antisera (T-absorbed) was absorbed with 0·35 mg of epidermal urea proteins only the epidermal basal cells stained (Fig. 7) as well as the papilloma and carcinoma (not shown). However, when 2 ml of the anti-carcinoma urea protein antisera was absorbed with 0·46 mg of epidermal urea proteins, epidermis was not stained (not shown), but papilloma and undifferentiated carcinoma fluoresced strongly. A level of 3 mg of epidermal urea antigens was required to absorb 2 ml of the T-absorbed anti-carcinoma urea protein antisera in order to remove the antibodies which stained both the carcinoma and papilloma (Table I); also absorption of 2 ml of the same

Fig. 6.—Papilloma of mice stained with T-absorbed anti-carcinoma urea protein antisera. ×200.

Fig. 7.—Basal cells of mouse epidermis were uniquely stained with T-absorbed anti-carcinoma urea protein antisera following absorption of 2 ml antisera with 0·35 mg of epidermal urea proteins. ×200.

antisera with 1·8 mg of papilloma or 0·52 mg of carcinoma urea proteins, respectively, resulted in a loss of staining of papilloma, carcinoma and epidermis.

The differences in the antisera raised against the urea proteins of normal epidermis and carcinoma as determined by fluorescence microscopy are summarized in Table I. The observations were obtained with many different samples of epidermis, papilloma and carcinoma with consistent results. It is apparent that by the proper absorption of T-absorbed anti-epidermal urea protein antisera with the carcinoma or papilloma urea antigens, antisera specific for epidermis can be prepared. Even though 2 ml of the T-absorbed and anti-epidermal urea protein antisera were absorbed with large levels of carcinoma (3·5 mg) and papilloma (4·5 mg) urea proteins, epidermis still stained strongly.

T-absorbed anti-carcinoma urea protein antisera stained the 3 related tissues intensely. By careful absorption of these antisera with epidermal urea proteins, only the basal cells of epidermis as well as the entire structure of the papilloma and carcinoma stained whereas further absorption with the epidermal urea antigens resulted in fluorescence only in the papilloma and carcinoma. Thus, T-absorbed anti-carcinoma urea protein antisera, did not distinguish between papilloma and carcinoma and both of these tissues, but not normal epidermis, stained following absorption of 2 ml of these antisera with 1·7 mg epidermal urea proteins. The antibodies which stained papilloma and carcinoma were removed gradually when anti-carcinoma urea protein antisera were absorbed with increasing levels of epidermal urea antigens, until at a high level of absorption with 3 mg epidermal urea proteins, the related tissues did not stain. However, absorption of the T-absorbed anti-carcinoma urea protein antisera with either papilloma or carcinoma urea antigens removed antibodies which were responsible for the fluorescence in epidermis, papilloma or carcinoma.

The urea proteins were also extracted from mouse undifferentiated squamous cell carcinoma, and antisera were prepared in rabbits against these proteins in the usual manner. Both the urea proteins from differentiated and undifferentiated carcinomata developed precipitin bands of identity in agar with antisera raised against either carcinoma proteins. Also, fluorescence microscopy did not reveal any differences between the T-absorbed differentiated and undifferentiated carcinoma antisera absorbed with undifferentiated and differentiated carcinoma urea proteins respectively. Since the undifferentiated carcinomata are relatively keratin free, fluorescence microscopy further indicates that the urea proteins are cellular derived. Antisera raised against the hyperplastic epidermal urea antigens reacted in a fashion somewhat similar to those of normal epidermis. Changes in the cell types (differentiating, resting, mitotic and resting in mitosis) in normal epidermis, papilloma and carcinoma (Glücksmann, 1945) did not appear to be related to the differences in the urea antigens between epidermis and papilloma or carcinoma.

The studies reported here on the urea proteins of epidermis, papilloma and carcinoma by fluorescence microscopy confirm the antigenic differences observed by immunodiffusion (Carruthers, 1970), and with the paired label technique (Bhattacharaya and Carruthers, 1972). The urea-extracted proteins of mammalian epidermis consist of several species as indicated by molecular sieve chromatography, and these components have a common antigenic determinant (Carruthers and Bhattacharaya, 1972*a*; Tezuku and Freedberg, 1972). The present studies, however, do not indicate the nature of the protein changes between epidermis and papilloma or carcinoma. There may be different proteins in epidermis than in papilloma or carcinoma even though the urea proteins of one of the related tissues can remove antibodies raised against any of the other 2 related tissues (Carruthers, 1970). Also, the 3 tissues have similar cell types

8 9

FIG. 8.—Precipitin patterns of 120 μg human epidermis (EP) and 120 μg squamous cell carcinoma urea proteins (CA) with 40 μl antisera (in centre well which was absorbed with pooled normal human serum, liver and kidney sediments) raised against EP.

FIG. 9.—Precipitin patterns of 120 μg human epidermis (EP) and 120 μg squamous cell carcinoma urea proteins (CA) with 40 μl antisera (in centre well which was absorbed as in Fig. 8) raised against CA.

(Glücksmann, 1945). The investigations of Rudall (1952) indicate that the fibrous or structural (urea-extracted) proteins of epidermis arise from the tonofilaments and are thus intracellularly derived. In this connection radiolabelled antibodies prepared against epidermal urea proteins did not penetrate epidermal cells *in vivo* (Bhattacharaya and Carruthers, 1972). Actually, Tezuka *et al.* (1972), have been able to reconstruct tonofilament-like structures *in vitro* from urea extracted proteins of newborn rat epidermis. These structures were similar in size and morphology to native epidermal tonofilaments.

*Immunodiffusion*

Only a limited amount of work was done on the urea-extractable proteins of human epidermis and squamous cell carcinoma; this was restricted to double diffusion in agar. Immunodiffusion patterns of the human epidermal and squamous cell carcinoma urea proteins when diffused against antisera prepared against the epidermal urea proteins are shown in Fig. 8. Two precipitin bands developed with the epidermal urea antigens but none were discernible with carcinoma urea antigens. When the same proteins were reacted with antisera raised against the carcinoma urea proteins, visible precipitin band formation was formed only against the carcinoma urea proteins (Fig. 9). Furthermore, the anti-carcinoma urea protein antisera gave a precipitin band of identity with the carcinoma urea 4·5 (proteins which are extracted with 6 mol/l urea and are precipitated maximally at pH 4·5) and 5·5 proteins, the identity of which is in accordance with the close relationship between these proteins (Carruthers and Bhattacharaya, 1972*a*). However, there was no reaction in agar with urea 5·5 proteins extracted from human breast carcinoma or oat cell carcinoma (lung) with the antisera raised against the squamous cell carcinoma urea proteins. These differences suggest the possibility of developing fluorescent-labelled antisera specific for squamous cell carcinomata and their detection in specific staining of tissue sections by fluorescence microscopy.

The anti-mouse carcinoma urea protein antisera gave strong precipitin bands with the human carcinoma urea proteins, but no discernible bands developed with the human epidermal urea proteins. Also, the anti-human carcinoma urea protein antisera gave weak precipitin bands with the mouse carcinoma urea proteins, but no visible bands developed with mouse epidermal urea proteins. Hence the carcinoma urea proteins of both species appear to have common antigenic determinants. The anti-mouse and anti-human epidermal urea protein antisera did not show a comparable cross-reactivity when reacted with human and mouse epidermal urea antigens respectively. However, Bauer (1972) has demonstrated that antibodies raised against the alkali 5·5 protein of human epidermis cross-reacted with the same proteins isolated from rabbit and bovine snout epidermis.

The author is indebted to Misses A. Baumler and A. Heining for the preparation of the urea-extractable proteins and for immunodiffusion, to Miss P. Weyland for the fluorescence microscopy, and to Dr U. Kim for the human squamous cell carcinomata.

This work was supported in part by Grant Number CA-07236 from the National Cancer Institute, National Institute of Health, U.S.A.

### REFERENCES

AVRAMEAS, S. & TERNYNCK, T. (1969) The Cross-linking of Proteins with Glutaraldehyde and its Use for the Preparation of Immunoadsorbants. *Immunochemistry*, **6**, 53.

BAUER, F. W. (1972) Studies on Isolated Keratin Fractions from Mammalian Epidermis. *Dermatologica*, **144**, 217.

BHATTACHARAYA, M. & CARRUTHERS, C. (1972) Antigenic Differences between Normal Mouse Epidermis and Methylcholanthrene-induced Squamous-cell Carcinomas. *Oncology*, **26**, 1.

CARRUTHERS, C. (1970) Urea-extractable Antigens in Normal, Benign and Neoplastic Mouse Epidermis. *Oncology*, **24**, 321.

CARRUTHERS, C. & BAUMLER, A. (1965) Immunochemical Staining with Fluorescein-labeled Antibodies as an Aid in the Study of Skin Cancer Formation. *J. natn. Cancer Inst.*, **34**, 191.

CARRUTHERS, C. & BAUMLER, A. (1966) Localization of Fluorescein-labeled Antibodies of Epidermal Proteins in Normal and Malignant Squamous Epithelium. *J. natn. Cancer Inst.*, **37**, 301.

CARRUTHERS, C. & BHATTACHARAYA, M. (1972*a*) Correlation between Various Proteins of Bovine Snout Epidermis. *Br. J. Derm.*, **86**, 495.

CARRUTHERS, C. & BHATTACHARAYA, M. (1972*b*) Antigenic Changes in Mouse Epidermis at Various Stages of Neoplastic Transformation. *Gann*, **63**, 299.

CARRUTHERS, C., WOERNLEY, D. L., BAUMLER, A. & KRESS, B. (1955) Proteins of Mammalian Epidermis. *J. invest. Derm.*, **25**, 89.

CARRUTHERS, C., WOERNLEY, D. L., BAUMLER, A. & LILGA, K. (1957) Studies on Certain Proteins in Normal and Pathological Epidermis. *Br. J. Cancer*, **11**, 597.

GLÜCKSMANN, A. (1945) The Histogenesis of Benzyprene-induced Epidermal Tumors in the Mouse. *Cancer Res.*, **5**, 385.

MASSEYEFF, R. & ZISSWILLER, R.-C. (1969) A Versatile Method of Radial Immunodiffusion Assay Employing Microquantities of Antiserum. *Analyt. Biochem.*, **30**, 180.

RUDALL, K. M. (1952) The Proteins of Mammalian Epidermis. *Adv. Protein Chem.*, **7**, 253.

TEZUKA, T. & FREEDBERG, I. M. (1972) Epidermal Structural Proteins. II. Isolation and Purification of Tonofilaments of the Newborn Rat. *Biochim. biophys. Acta*, **263**, 382.